# Probiotics Strains Modulate Gut Microbiota and Lipid Metabolism in Mule Ducks

**DOI:** 10.2174/1874285801812010071

**Published:** 2018-04-23

**Authors:** Maxime Even, Stéphane Davail, Mikael Rey, Annabelle Tavernier, Marianne Houssier, Marie Dominique Bernadet, Karine Gontier, Géraldine Pascal, Karine Ricaud

**Affiliations:** 1 UMR 1419 INRA UPPA NuMéA, 371 rue du ruisseau, 40000 Mont de Marsan, France; 2 UMR 1419 INRA UPPA NuMéA, Quartier Ibarron, 64310 Saint Pée sur Nivelle, France; 3 UEPFG INRA Bordeaux-Aquitaine, (Unité Expérimentale Palmipèdes à Foie Gras), Domaine d’Artiguères 1076, route de Haut Mauco, F-40280 Benquet, France; 4 GenPhySE, Université de Toulouse, INRA, INPT, ENVT, Castanet-Tolosan, France

**Keywords:** Duck, Intestinal microbial diversity, Overfeeding, Probiotics, Lipid metabolism, Immune gene expression

## Abstract

**Background:**

Livestock production should respond to societal, environmental and economic changes. Since 2006 and the ban on antibiotics as growth factors in European Union, the use of probiotics has become widespread and has demonstrated the effect of intestinal microbiota on the performance of farm animals.

**Objective:**

The aim of this study was to investigate the effect of supplementation with *Lactobacillus salivarius* (as a probiotics strain or combined with other strains) on zootechnical performance, metabolic and immune gene expression and intestinal microbiota diversity in mule ducks using high-throughput sequencing and real-time PCR.

**Method:**

The mule ducks were reared for 79 days and overfed for 12 days with or without probiotics. Samples were collected at 14 (starting period) and 91 days (end of overfeeding period), 3 hours post feeding.

**Results:**

Irrespective of digestive content, age, level of feed intake or supplementation with probiotics, *Firmicutes*, *Proteobacteria* and *Bacteroidetes* were the dominant phyla in the bacterial community in mule ducks. At 14 days, both the ileal and cecal samples were dominated by *Firmicutes* (in particular the *Clostridiales* order). Overfeeding induced a shift between *Clostridiales* and *Lactobacillales* in the ileal samples whereas in the cecal samples, the relative abundance of *Firmicutes* decreased. Overfeeding also induced hepatic over-expression of Fatty Acid Synthase (*FAS*) and of the lipid transporter gene Fatty Acid Binding Protein 4 (*FABP4*). This increase in lipid metabolism genes is associated with a decrease in inflammatory response.

**Conclusion:**

Finally, probiotic supplementation had only a slight impact on gene expression and microbiota diversity, both at 14 days and after overfeeding.

## 1 INTRODUCTION

The intestinal microbiota plays an essential role in the host physiology and forms a complex ecosystem [1]. Microbiota can affect gut morphology or nutrient utilization [2], stimulate the immune response [3] and protect against pathogens [4].

In chickens, *Lactobacillus* spp. is the major genus in the small intestine (duodenum, jejunum and ileum) whereas *Clostridium* spp. and *Bacteroides* spp. are dominant in the ceca [5, 6]. In ducks, *Firmicutes* and *Bacteroidetes* are the dominant phyla in both the ileum and the ceca, as previously described in chickens and mammals [7-10]. However, *Lactobacillus* is not dominant, as described in adult chickens [8, 9]. In rearing geese, the dominant phyla are *Proteobacteria* and *Firmicutes* in intestinal microbiota [10, 11]. Previous studies have also shown that the diversity of intestinal microbiota is modified by diet [12]. During overfeeding, waterfowls are exclusively fed with corn which induces a hepatic steatosis called “foie gras”, resulting from the storage of fatty acids in the liver [13, 14]. Recent studies have shown a strong effect of diet and genetics on microbiota diversity, in particular an increase in *Lactobacillus* spp. after overfeeding [8-10]. Interestingly, a link between the ability of ducks to trigger a liver steatosis and the composition of the intestinal microbiota according to the genetic type has also been highlighted [8, 9].

Probiotics (live microorganisms) are known to be beneficial to their host [15] and since the ban on the use of antibiotics as growth factors in European Union, their use to improve animal health, welfare and productivity has increased [16]. Various actions have been described; for example, probiotics can reduce the presence of pathogenic bacteria in the intestine, probably through the production of lactic acid, bacteriocins or both [4, 17, 18]. Competition can also occur for the occupation of ecological niches [19]. Immune resistance has also been modulated by probiotic supplementation [20]. Probiotics have an effect on body weight gain and liver weights in young chicks and ducklings [21]. In broiler chickens, Kabir *et al.* [22] demonstrated a higher liver weight and better immune to sheep red blood cell immunization when chickens were supplemented with *Enterococcus faecium*. The *Lactobacillus* genus has also been regularly cited as an efficient probiotic in poultry [23, 24]. *Lactobacillus* is known to have hydrolytic action and to favor digestion and absorption of nutrients, making them available to the host [25, 26]. *Lactobacillus* inoculation in young chicks and ducklings has an effect on body weight gain and liver weights [21]. It has also been shown in geese that anti-inflammatory immunity mechanisms are linked to the *Lactobacillus* genus, and partially explain the animal's good health during overfeeding [10]. While many studies have been carried out on the effect of microbiota on poultry performance [27, 28], little is known about the role of microbiota in waterfowl during overfeeding.

The rapid evolution of the diversity of intestinal microbiota during the early stages of development demonstrated by Best *et al*. [29] and Rey *et al*. [30] in ducks and the strong effect of strain inoculation in newborn chickens on the composition of microbiota [31, 32] suggest that supplementation in ducklings will be more efficient in modulating microbiota and host physiology immediately after hatching.

In the poultry industry, there is an increasing interest in probiotics because in many countries, growers can no longer use antibiotics as growth factors [33]. In this study, we analyzed the effect of probiotic supplementation (*Lactobacillus salivarius* isolated from ducks at the end of overfeeding or strains isolated from chickens) on growth performance, microbial diversity, metabolism and immune gene expression in ducks.

## 2 MATERIALS AND METHODS

### 2.1 Animals and Experimental Design

This trial was performed from February 2^nd^ 2015 to May 4^th^ 2015. The animals were cared for in accordance with the animal research guidelines of the French Ministry of Agriculture and the Directive 2010/63/EU. This trial was carried out at the Experimental Station for Waterfowls of INRA (Benquet, France with accreditation number B40-037-1).

The schematic diagram of the experimental design is available in Fig. (**1**). A total of 150 post-hatching ducklings were randomly allocated to three separate pens (18m^2^ per pen). The first group received the probiotic isolated by the cultural method (group A). The second received a probiotic product composed of a mix of strains isolated from healthy chickens (group B). Sterilized water was used as a control for the third group (group C).

All ducks were fed *ad libitum*, from hatching to 28 days of age, with a starting diet (11.43 MJ/kg; crude proteins: 17.5%) and from 28 to 79 days of age with a growing diet (11.37 MJ/kg; crude proteins: 15.5%). At the age of 56 days, the animals were subjected to hourly rationing in order to prepare the overfeeding. At 80 days of age, these ducks were overfed with corn mixture (13.9 MJ/kg; crude proteins: 8.9%) for 12 days in cages containing four ducks (21 meals). During the overfeeding period, the ducks were allocated to cages according to the experimental group. The composition of the different diets used in this study is listed in (Table **1**).

During the rearing period (from hatching to 79 days of age), it was decided to mix the feed with adequate probiotics (group A and B) up to a final bacterial load of 2.10^8^ CFU / g; or with sterile water (group C). During the overfeeding period (from 80 to 91 days of age), the animals were inoculated with probiotic A or B or sterile water by oral gavage with a gastric tube and syringe before the meal, according to the weight of diet received by the animal. During the overfeeding period, the animals that had been given probiotics during the rearing period were divided into two equal subgroups. One of the two subgroups continued to receive the adequate probiotics (group A+ and B+) and the other did not (group A- and B-), instead receiving sterile water like the control animals (group C). The individual BW (body weight) was registered every 14 days during experimentation. Feed distribution was registered every 2 days during the period from day 1 to day 79 (individual pen measurements). Food intake was measured at each distribution by deducting uneaten food from distributed food. Food intake was registered individually during the overfeeding period (from 80 to 91 days of age). The ducks were killed by exsanguination after electric stunning, 3 h after the last meal to homogenize the filling level of the ducks’ digestive tract. Ten animals from each experimental group were selected at 14 days of age and were considered as the control group for the overfeeding effect (starting point, SP point). Ten animals from each experimental group were selected at 78 days of age and were only considered for their performance level (before overfeeding, Bof point). The 30 remaining animals were killed at the end of the overfeeding period (End of Overfeeding, Eof point).

### 2.2 Probiotics and Food Preparation

Two probiotics were used in this experiment. The first one (A) was a *Lactobacillus* strain isolated by the cultural method on MRS/Rogosa media (VWR chemicals, Radnor, Penn, USA) from the ileal digestive content of a duck at the end of overfeeding. Ileal digestive contents were an collected and inoculated on MRS/Rogosa agar plate after serial dilution in a Tryptone buffer. Ten characteristics colonies were purified on the MRS/Rogosa. Once isolated and purified, these strains were cultured in liquid MRS medium for 12 h at 37° C. These strains were then identified by sequencing the PCR product of the gene encoding the 16S RNA (Custom DNA Sequencing, Eurofins Genomics, Luxembourg, Luxembourg). Three strains were identified as *Lactobacillus salivarius*. The cultures of these three strains were then centrifuged to pellet the cells and resuspended in sterile water with a ratio of 0.8-fold the volume of culture. The stability of resuspended cells in the animal food was tested by mixing the resuspended cells with feed to a bacterial load of 2.10^10^ CFU / g. The most stable strain was selected. Bacterial quantification decreased from 2.10^10^ to 2.10^8^CFU / g during the first 24 h after diet preparation, so diets with probiotics were prepared and distributed 24 h later during the animal experimentation. The second probiotic is a probiotic product in powder form including probiotic bacteria isolated from the small intestinal tract (*Enterococcus faecium* and *Bifidobacterium animalis*) and cecum (*Pediococcus acidilactici* and *Lactobacillus salivarius*) of healthy adult chickens. The product had a total bacterial count of 1.10^11^ CFU / g. To inoculate the animals, it was decided to mix probiotic B with the diet, at the same dose as probiotic A. For the control group, the same volume of sterile water was used.

### 2.3 Sampling for Biological Analysis

Blood was sampled for plasma analysis. The plasmas were separated by centrifugation at 3000×*g* for 10 min at 4° C and stored at −20° C. After dissection, the liver, *pectoralis major* (muscle) and subcutaneous adipose tissues (SAT) were weighed, sampled and stored at −80° C for the study of gene expression. The ileum and ceca were immediately collected and kept on ice. The digestive contents of the ileum and ceca were collected by gently squeezing the organ and stored at −80° C for the study of gut microbiota.

### 2.4 Fatty liver Melting Rate Measurement

In order to determinate the fatty liver melting rate (or fat loss during cooking), approximately 200g of the fatty liver were weighed and put into a glass jar with salt (12g/kg) and pepper (2g/kg). The jars were then cooked for 1 h in water in an autoclave at 85°C under a pressure of 0.8 bar. The temperature was controlled in water and in control jars equipped with temperature sensors. After 30 min of chilling by circulating cool water in the autoclave, the jars were stored at 4°C. The jars were opened after 2 months and exuded fat during cooking was carefully removed from the liver. The fatty liver melting rate was evaluated as: $\frac{\mathrm{cooked}\;\mathrm{fatty}\;\mathrm{liver}\;\mathrm{weight}\;(\mathrm{trimmed}\;\mathrm{of}\;\mathrm{loose}\;\mathrm{fat})}{\mathrm{raw}\;\mathrm{fatty}\;\mathrm{liver}\;\mathrm{weight}}\text{∗}100$

### 2.5 Biochemical Assays

Plasma Glycemia and Triglyceridemia were quantified by the colorimetric method using enzymatic kits (Glucose RTU, Biomérieux, France and Triglycérides LDB, Biodirect, France).

### 2.6 Gene Expression Measurement

#### 2.6.1 RNA Isolation and Reverse Transcription

Total RNA was isolated from the frozen tissue according to the TRIZOL method (Invitrogen/Life technologies, Carlsbad, Cal, USA). Total RNA concentration was measured by spectrophotometry using a NanoVuePlus (GE Healthcare, Chicago, Illinois, USA), and all samples were normalized at 500 ng /µl before storage at -80°C until cDNA generation. The integrity of total RNA was analyzed by electrophoresis. cDNA was obtained by reverse transcription using the enzyme Superscript III (Invitrogen/Life technologies, Carlsbad, Cal, USA) and a mix of oligo dT and random primers (Promega, Fitchburg, Wisconsin, USA). 3 µg of total RNA was used, and the absence of contamination by DNA was verified by two negative controls (without RNA and without Superscript). The reaction was carried out in a StepOne (Applied Biosystem, Waltham, Massachusetts, USA) for 25° C / 5 min, 55° C / 60 min, 70° C / 15 min and retention at 4°C until storage at −20°C.

#### 2.6.2 Real Time PCR, Primers and Gene Expression Analysis

Ten birds per experimental group (with or without probiotics according to sampling point) were used for quantitative PCR analysis, both for metabolism and immune response gene expression. All primer sets are listed in Table (**2**). The reactions were run in duplicate in a final volume of 15 μL. The PCR mix was made up of 7.5 μL of SybrGreen Universal PCR Master Mix (Quanta Bioscience, Gaithersburg, MD, USA), 5.5 μL of 500 nM specific primers, and 2 μL of template cDNA or the negative controls. Real-time PCR was performed in a StepOne instrument (Applied Biosystem, Waltham, Massachusetts, USA) with an initial denaturation step of 10 min at 95° C, and 35 cycles for denaturation of 15 s at 95° C, annealing/extension for 1 min at a specific temperature for each primer, and 1 final cycle at 70° C for 15 s. Melt curve analyses were done by slowly heating the PCR mixtures from 60 to 95° C, and the cycle threshold (Ct) was determined with the StepOne Applied Biosystem software 2.3. The chosen reference gene was Actin B but all the results were confirmed with glyceraldehyde 3-phosphate dehydrogenase (GAPDH). The standard group was group C at SP point. For several genes, results could only be obtained at Eof point. In this case, the reference group was group C at Eof point. All Cycle thresholds (Ct) were collected. Results are expressed as 2^-∆∆Ct^ with ∆∆Ct= ((Ct_target_ - Ct_ref_) sample)- ((Ct_target_- Ct_ref_) standard).

### 2.7 Metagenomic Sequencing of Intestinal Microbiota

#### 2.7.1 DNA Extraction

Total genomic DNA from the ileal and cecal samples was extracted by combining mechanical and thermic lysis using an Ultra Turrax Digital Homogenizer IKA T-25 (Fisher Scientific, Illkirch, FR) and a QIAamp Fast DNA Stool Mini Kit (QiagenGmbh, Hilden, DE) according to the manufacturer’s instructions with 220mg as starting material. The lysis temperature used was 95°C. The DNA sample was eluted with 50 µl of ATE buffer (Qiagen Gmbh, Hilden, DE) and stored at – 20 °C. The quantity and quality of DNA extracted were measured using the NanoVue Plus (GE Healthcare, Chicago, Illinois, USA).

#### 2.7.2 16S rRNA Amplification and Sequencing

The PCR for sequencing were realized on the 16S rRNA gene according to the method described by Lluch *et al*. [34] using MiSeq kit reagents v2 (2x250 bp pair ended reads). Amplicons from the V3-V4 regions of 16SrRNA genes were generated using specific bacterial primers 5’CTTTCCCTACACGACGCTCTTCCGATCTAC GGRAGGCAGCAG 3’ and 5’GGAGTTCAGACGTGTGCT CTTCCGATCTTACCAGGGTATCTAATCCT 3’. The preparation of amplicons was performed in a total volume of 50µL containing 1 U TAQ Polymerase and adequate 10 X PCR buffer (MTP Taq DNA Polymerase, Sigma), 200µM of dNTP (Sigma), 0.2µM of each primer and 2µL of DNA template. The amplification program consisted of an initial denaturation step at 94°C for 1 min and 32 cycles of denaturation at 94°C for 1 min, annealing at 63° C for 1 min and elongation at 72° C for 1 min. In the end, a final extension step at 72° C for 10 min was carried out. The quality of PCR products was controlled by electrophoresis. 2µL of PCR product were deposited on an agarose gel (1% / TBE) with load Buffer for 30- 40 min at 135 V. Amplicons were then sent to the INRA genomic platform in Toulouse for sequencing. The amplicons were purified briefly using the magnetic beads Agencourt AMPure XP- PCR Purification (Beckman Coulter, Brea, CA, USA) following the 96-well format procedure, modified as follows: beads / PCR reactional volume ratio of 0.8 x and final elution volume of 32 μl using Elution Buffer EB (Qiagen). The concentration of the purified amplicons was controlled using Nanodrop 8000 spectrophotometry (Thermo Scientific). Single multiplexing was performed using a homemade 6-bp index, added to reverse primer during a second PCR with 12 cycles using a forward primer (5’AATGATACGGCGACCACCGAGA TCTACACTCTTTCCCTACACGAC 3’) and reverse primer (5’CAAGCAGAAGACGGCATACGAGAT-index-GTGACTGGAGTTCAGACGTGT 3’). The resulting PCR products were purified and loaded onto the Illumina MiSeq cartridge according to the manufacturer’s instructions. The quality of the run was checked internally using PhiX Illumina, and then each pair-end sequence was assigned to its sample with the help of the previously integrated index.

### 2.8 Sequencing Analysis

All software for analysis of sequenced data was used in the FROGS pipeline developed by the French National Institute of Agricultural Research (INRA, Toulouse, France) [35].

First the sequences were denoised using the Flash 1.2.11 and Cutadapt 1.7.1 tools [36, 37]. Only sequences with sizes between 380 and 500 bp, without ambiguous bases and with the two primers (5’ primer ACGGGAGGCAGCAG and 3’ primer AGGATTAGATACCCTGGA) were kept. Secondly, the sequences were clustered with Swarm 2.0 [38] in two steps, as recommended by Escudié *et al.* [35]. Swarm uses an iterative growth process to cluster sequences. In each growth step, the sequence of the previous step was used to find the other sequences with a number of differences inferior or equal to the “Aggregation distance”. The first step, or denoising step, served to build very fine clusters with an aggregation distance equal to 1 between the sequences of each crown. After the denoising, a second swarm with an aggregation equal to 3 between seeds from this first clustering was used to delineate OTUs. Next, PCR chimeras were removed using VChime of the Vsearch package [39]. This step was performed after clustering in order to shorten the analysis time. Next, the remaining clusters were filtered. Only clusters present in at least 2 samples and with an abundance greater than 0.005% of the total sequences [40] were kept. Finally, taxonomic affiliation for each OTU was obtained with BLASTn+ [41] by blasting the sequences on the Silva128 database [42].

### 2.9 Statistical Analysis

Values given in the text are expressed as means ± SEM. Analyses of the OTU table were performed with the PhyloSeq package [43]. Relative abundance, Chao1 diversity richness, Shannon and InvSimpson richness were determined. Beta-diversity was determined using nMDS with the Bray-Curtis distance method. Sparse Partial Least Squares Discriminant Analysis (s PLS DA) was performed to determine the most discriminant OTU using the mixOmics package [44] with CSS normalization and log transformation of count sequences. A cutoff value of 0.95 was used to select the discriminant OTUs. Statistical analyses were performed with ANOVA analysis of variance using the R software 3.3.1 [45]. Differences were considered as significant when P-value < 0.05. When significant, differences between treatments were compared using Tukey’s test and FDR with BH correction [46]. In order to analyze relationships between the bacterial community and bio-chemical plasma parameters and growth parameters, an sPLS (sparse Partial Least Squares) analysis was also carried out using the mixOmics package.

## 3 RESULTS

### 3.1 Bird Performance

Concerning group C, during the rearing period, BW increased from 508±6 g (SP point) to 4306±75 g (Bof point) (*P*<0.001). Moreover, BW was statistically different between experimental groups at SP point. In fact, the BW of group C at SP point was higher than those of group A and B at the same point (508±6 g *vs* 486±5 and 484±6 g; *P*<0.001). This difference disappeared at 42 days of age (data not shown). During the rearing period, no effect of probiotic addition was observed on liver weight (LW), muscle weight (MW) and subcutaneous fat weight (SFW). In group C, between SP and Eof point, the overfeeding period increased BW (4 306±75 g to 6 261±97 g; *P*<0.001), LW (93±5 g to 628±21 g; *P*<0.001) and SFW (57±8 g to 151±5 g; *P*<0.001). MW was not influenced by overfeeding (310±13 g *vs* 315±5 g; N.S) Table (**3**). As previously, no effect of probiotic addition during overfeeding was observed on BW, LW, MW and SFW.

Feed consumption during overfeeding was expressed as g of raw feed/bird Table (**3**). No statistical difference in feed consumption was observed during rearing (data not shown) and the overfeeding period Table (**3**), with or without probiotic supplementation. The fatty liver melting rate (fat loss during cooking) was used to determinate fatty liver quality. Here, probiotic addition had a significant effect on the fatty liver’s melting rate. In fact, the melting rate decreased in group A- at Eof point compared to group C at the same point (19.6±1.2% *vs* 12.7±1.7%; *P*<0.05) (Table **3**). However, no significant differences were observed between group A- and the other probiotic groups (A+, B-, B+).

### 3.2 Glycemia and Triglyceridemia

No effect of probiotic supplementation was observed during SP point on glucose and triglyceride plasma concentration Table (**3**). Between SP and Eof point, glycemia increased (from 1.99±0.71 g / L to 2.94±0.84 g / L in group C; *P*<0.005), as did triglyceridemia (from 1.43±0.25 mmol / L to 4.76±1.37 mmol / L in group C; *P*<0.001). Probiotic supplementation had no impact on glucose concentration at Eof point. Concerning the plasma concentration of triglycerides during Eof point, we observed that group A+ had a higher triglyceridemia than group C (5.86±1.32 *vs* 4.76±1.37 mmol / L; *P*<0.05). However, no significant differences were observed between group A+ and the other probiotic groups (A-, B-, B+).

### 3.3 Metabolic and Immune Response Gene Expression

The expression of gene markers of lipogenesis and lipid uptake were then highlighted Table (**4**). Metabolic gene expression was measured in the liver. Fatty Acid Synthase (*FAS*) expression increased strongly between SP and Eof point (717±312-fold in group C; *P*<0.005). In the same way, Fatty Acid Binding Protein 4 (*FABP4*) expression increased strongly between SP and Eof point (177±77-fold in group C; *P*<0.001). However, probiotic supplementation had no effect on metabolic gene expression either at Eof point or at SP, irrespective of the tissues and genes studied Table (**4**). For Fasting-Induced Adipose Factor / angiopoietin-like protein 4 (Fiaf factor), expression increased more than 3 times between SP and Eof point.

Immune response gene expression was measured in the jejunum intestinal mucosa. Lipopolysaccharide induced TNFα (*Litaf*) expression decreased between SP and Eof point (0.42±0.09-fold the expression in group C; *P*<0.05). Moreover, Interleukin 8 (*IL8*) expression was only detected at SP point and not at Eof point. Conversely, *PPARγ* expression was up-regulated at Eof point (4.8±0.4-fold in group C; *P*<0.05). Finally, probiotic supplementation had no impact on the expression of immune response genes at SP point. However, at Eof point, expression of *Litaf* was lower in group B+ than groups A+, A- and C but not significantly different from group B-.

### 3.4 Bacterial Community

A total of 4,603,890 16s RNA sequences were obtained from the MiSeq sequencing for the 102 samples. After pre-processing with FROGS, a total of 4,029,552 sequences were kept. After clustering with Swarm 2.0, we obtained 356,437 clusters. Then the PCR chimeras (70,343 clusters), representing 190,937 sequences, were removed. The 286,094 remaining clusters were filtered according to several criteria and the clusters present in at least 2 samples were kept as well as the ones representing at least 0.005% of total sequences [40]. Thus, 412 clusters or OTUs (Operational Taxonomic Units) were kept, representing 75.9% of total sequences, with an average of 104±5 OTUs per sample representing an average number of sequences of 33,321±1037 per sample. Finally, 7 Phyla, 99 genera and 412 species were detected in all samples independently of experimental group or sample origin.

#### 3.4.1 Ileal Bacterial Community (Core Microbiota in all Samples)

In the ileal content, the major phylum, independently of the experimental group, was *Firmicutes* (98.3±1.4%). *Proteobacteria* represented around 1.4±0.5% of the population. Finally, *Bacteroidetes* and *Actinobacteria* each represented 0.1±0.1% of the population. Other phyla such as *Tenericutes* and *Fusobacteria* represented less than 0.1% of the population Fig. (**2a**). To evaluate the microbiota composition at finer taxonomic levels, order distributions were analyzed Fig. (**2**). The phylum of *Firmicutes* was dominated by *Clostridiales* and *Lactobacillales* orders (55.6±9.9% and 42.7±7.3% respectively of the total population) and *Proteobacteria* was dominated by the orders of *Burkholderiales* (0.6±0.5% of the total population) and *Enterobacteriales* (0.2±0.1% of the total population). A representation of ileal microbiota on family level according to experimental group is available in Fig. (**3**). Finally, 95 genera were detected in all ileal samples.

#### 3.4.2 Cecal Bacterial Community (Core Microbiota)

In the cecal content, the major phyla, regardless of the experimental group, were *Firmicutes* (50.6±17.4% of the total population), *Bacteroidetes* (25.3±10.2% of the total population) and *Proteobacteria* (23.4±9.4% of the total population). *Actinobacteria* and *Tenericutes* represented around 0.6±0.5% of the population. Finally, *Fusobacteria* and *Cyanobacteria* represented less than 0.1±0.1% of the population Fig. (**4a**). At order level, *Firmicutes* were dominated by *Clostridiales* (47.7±16.5% of the total population) and *Bacteroidetes* were dominated by *Bacteroidales* (25.2±10.2% of the total population). The orders of *Enterobacteriales* and *Desulfovibrionales* accounted for 10.6±4.4% and 12.0±5.0% of the total population for the *Proteobacteria* phylum Fig. (**4b**). A representation of cecal microbiota on family level according to experimental group is available in Fig. (**5**). Finally, all 99 genera were detected in all cecal samples.

### 3.5 Effect of Overfeeding and Probiotics on Ileal and Cecal Microbial Communities in Mule Ducks

**Fig. (1):**
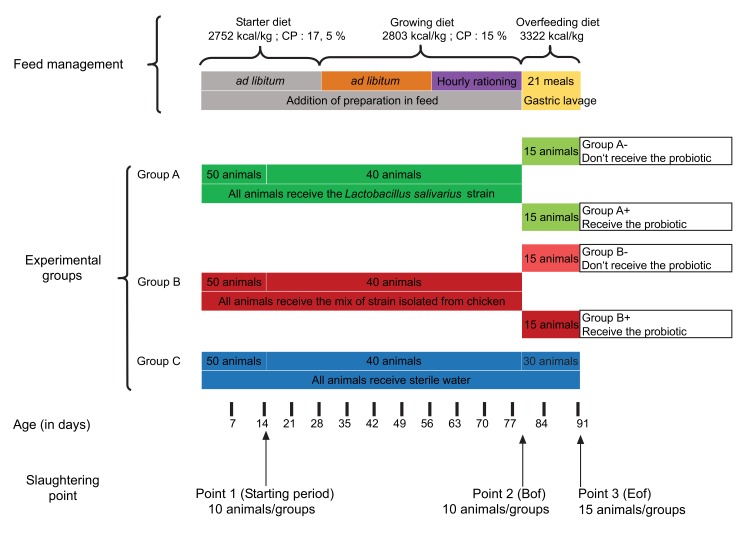
Schematic diagram of the experimental design. During the starter and growing diets, animals from each experimental group were reared in separate pens (18m2 per pen). Heating was provided for the first 14 days, then no heat was provided and the animals had access to the outdoor pen (8m2).

**Fig. (2):**
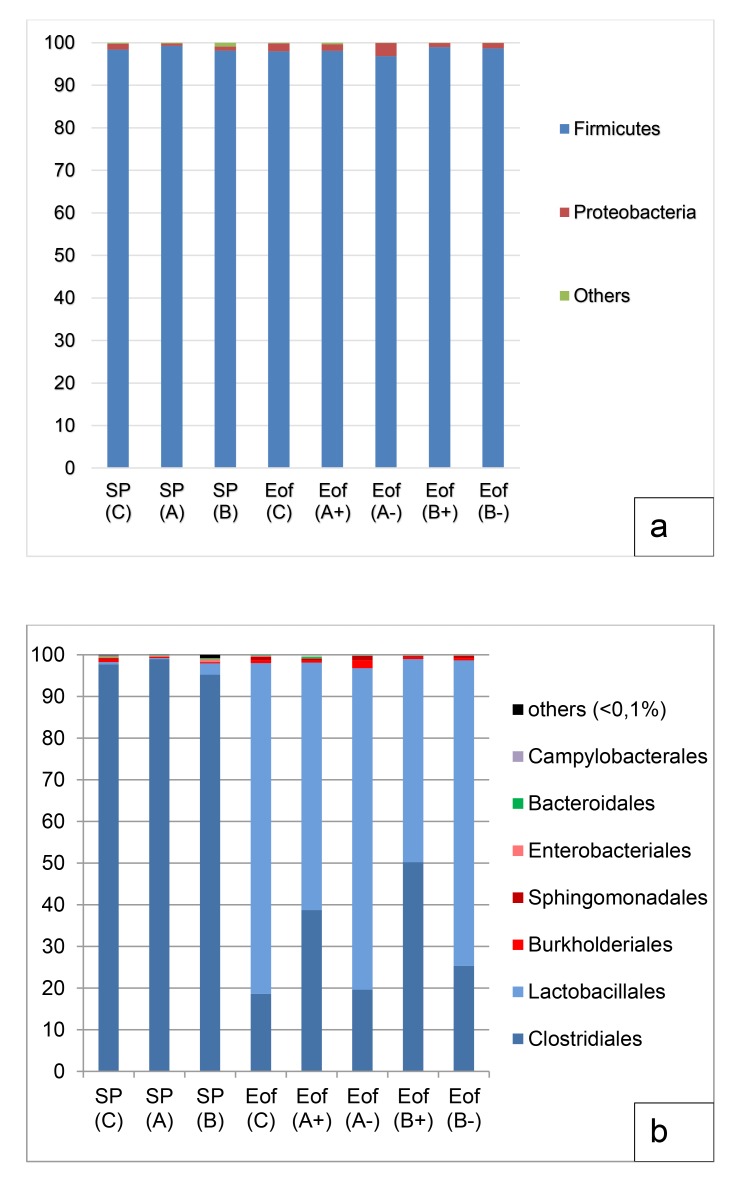
Relative abundance (%) evaluated at the phylum (a) and order (b) levels of main bacterial groups in the ceca before (Bof) and after (Eof) overfeeding according to experimental group.

In both the ileal and cecal samples, probiotic addition had no effect on richness and diversity at SP point, but at Eof, diversity and richness tended to decrease Table (**5**). However, in group C, the Shannon index was lower at SP point than all groups at Eof point (0.5±0.1 to 1.8±0.1 in group C at EOF point; *P*<0.005) in the ileal samples. In the same way, no statistical difference was observed in the cecal samples between SP and Eof point in the number of observed OTUs and Chao1 index Table (**5**). However, in group C, InvSimpson in ceca tended to decrease at Eof point compared to SP point (17.2±2.7 in to 6.9±3.1; N.S). This difference was statistical between both groups A at SP point and groups A at Eof point (30.0±5.0 to 7.8±2.3 in group A+ and 7.8±2.3 in group A-; *P*<0.05) and between groups B at SP point and groups B at Eof point (27.0±6.2 to 7.7±2.00 in group B+ and 9.1±2.0 in group B-; *P*<0.05). Finally, during the rearing period probiotic addition had no effect on either the ileal or the cecal samples (Figs. **1** and **2**).

#### 3.5.1 Effect on Ileum

No effect of probiotics or overfeeding was detected at phyla levels in the ileal samples. At order level, in group C, we observed a shift of *Clostridiales* (97.7±0.3% to 17.6±5.4%; *P*<0.001) and *Lactobacillales* (0.5±0.1% to 80.4±5.5%; *P*<0.001) between SP and Eof point. In the same group, the relative abundance of *Bacillales* also decreased between SP and Eof point (0.1±0.1% at SP point to 0.0±0.0% at Eof point; *P*<0.05).

In the same way, the probiotic addition had a slight effect on the order level. Group A- at Eof point had higher relative abundance of *Burkholderiales* (2.0±0.8%) than group A (0.2±0.1%; *P*<0.05) and group B (0.2±0.0%; *P*<0.05) at SP point. However, no statistical difference was observed between the other groups.

In the ileal samples, the *Lactobacillus* genus represents 0.1±0.1, 0.2±0.2 and 0.3±0.1% of the total population respectively in group A, B and C at SP point. No statistical difference was observed between experimental groups at SP point. In group C, as in the other experimental groups, the overfeeding period increased the relative abundance of the *Lactobacillus* genus (from 0.3±0.1 to 78.0±10.3% of total population, *P*<0.001). Regardless of the experimental group, the most abundant *Lactobacillus* during the rearing period were *L. aviarius, L. salivarius and L. amylovorus* with 0.1±0.1% of total relative abundance each. During the overfeeding period, the most abundant *Lactobacillus* species were *L. amylovorus* (9.8±7.2% of relative abundance) and *L. plantarum* (9.3±6.8% of relative abundance). During the overfeeding period, *L. salivarius* represented 1.5±0.5% of the *Lactobacillus* genus.

#### 3.5.2 Effect on Ceca

At phylum and order level, the probiotic addition had no impact on relative abundance at SP point. At phylum level, in group C, *Firmicutes* decreased from 99.1±0.2% at SP point to 17.2±7.6% of the population at Eof point (P<0.001). In the same way, in group C, *Actinobacteria* decreased between SP and Eof point (P<0.05). However, this phylum represented less than 0.1% of the total population. The decrease in the *Firmicutes* and *Actinobacteria* phyla between SP and Eof point was detected in all experimental groups independently of the supplementation of probiotics

On the other hand, *Bacteroidetes* and *Proteobacteria* tended to increase (respectively from 0.1±0.1% at SP point to 33.5±10.9% of population at Eof point and 0.6±0.2% to 48.6±16.4% of population, N.S) in group C. The increase in *Bacteroidetes* between SP and Eof point was significant between group A at SP point (0.1±0.1% of total population) and group A+ (50.3±9.8% of total population) (*P*<0.05). In the same way, the *Proteobacteria* phylum increased between group B (0.7±0.2% of total population) and group B+ (46.9±8.6% of total population) (*P*<0.05). At order level, in group C, the huge decrease in *Firmicutes* can be explained by the decrease in the *Clostridiales* order from 89.5±4.5% at SP point to 17.1±7.6% of total population at Eof point (*P*<0.001). In the same way, the increase in *Bacteroidetes* between group A at SP point and group A+ at Eof point was related to an increase in *Bacteroidales* (0.1±0.1% in group A to 50.3±9.8% of total population in group A+; *P*<0.05).

Finally, probiotic addition influenced the relative abundance at Eof point of *Campylobacterales* only, which decreased in group B+ (0.1±0.0% of total population; *P*<0.05) and group B- (absence of detection) compared to group C at Eof point (0.3±0.3% of total population; *P*<0.05). No statistical difference was observed between Eof point of groups B+ and B-. Conversely, an increase in *Campylobacterales* from 0.3±0.3% to 2.1±0.9% (*P*<0.005) was observed between group A+ and group C at Eof point. No statistical difference was observed between the other groups. At the species level, *Campylobacter jejuni* was dominant.

### 3.6 Determination of Discriminant OTUs

#### 3.6.1 s PLS DA for Ileal Samples

Discriminant analysis (s-PLS DA) was performed to identify the most discriminant OTUs between experimental groups. A first s-PLS DA in order to highlight the most discriminant OTUs was performed between SP and Eof point, independently of the probiotics supplementation Fig. (**6a**). The OTUs enabling to discriminate between these two groups were correlated to the OTUs affiliated to two *Lachnospiraceae* families (from the *Clostridiales* order and undetermined species) and to OTU_456 (affiliated to *Enterococcus durans* 100%). The second s-PLS DA was performed between the experimental groups at SP point Fig. (**6b**). Group B was the most separated group. The separation between these three groups was also correlated to 4 OTUs affiliated to *Lachnospiraceae*, 3 OTUs affiliated to *Ruminococcaceae* families (from the *Clostridiales* order and undetermined species) and to OTU_456 (affiliated to *Enterococcus durans* 100%). Finally, a third s-PLS DA was performed between the experimental groups at Eof point Fig. (**6c**). Groups A+ and B+ were the most variable groups. The separation between these two groups was correlated to 2 OTUs affiliated to *Lachnospiraceae* (undetermined species), 3 OTUs affiliated to *Ruminococcaceae* families (undetermined species) and to 3 OTUs affiliated to the *Lactobacillus* genus (OTU_400: *L. curvatus 98.5%*, OTU_44: *L. vaginalis 100%* and OTU_270: *L. paralimentarius 99%*). However, these OTUs are very minor (less than 0.05% of relative abundance).

#### 3.6.2 sPLS Analysis: Relationship Between Bacterial Taxonomic Profiles and Liver Bio-Chemical Parameters and Growth Performances

We studied the relationships between the bio-chemical plasma parameters and growth parameters and the OTU profile of the microbiota by sPLS. According to the leave-one-out process, to compute the Mean Square Error of Prediction, two components were computed and one projection plan (components 1 and 2) was considered. Fig. (**7**) is a graphical representation of the selected OTUs on the first two sPLS dimensions. The selected OTUs and the bio-chemical and growth parameters are projected onto correlation circles where highly correlated variates cluster together (within a data set or between the two data sets). The first component did not allow us to group together growth and bio-chemical parameters. Considering the OTUs, high number of them were negatively correlated with bio-chemical and growth parameters. Most of them were members of *Clostridiales*, especially *Lachnospiracae* and *Ruminococcae*, already highlighted to be the most discriminant OTUs between experimental groups in sPLS-DA.

#### 3.6.3 s PLS DA for Cecal Samples

Discriminant analysis (s-PLS DA) was also performed to identify the most discriminant OTUs between experimental groups. The first analysis was performed between SP and Eof point independently of the probiotics supplementation Fig. (**8a**). The OTUs enabling to discriminate between these two groups were at least correlated to OTU_230 affiliated to the *Alistipes* genus 97.6% (*Bacteroidales* order and undetermined species) and to OTU_431 (affiliated to *Clostridiales* vadin BB60 group 97.7%). It is nevertheless noteworthy that the increase in the *Alistipes* between the two points was significant (from 0.0±0.0 to 6.8±2.3% of total population in group C up to 10.2±3.2% in group A-; *P*<0.005). A second s-PLS DA was performed at SP point for groups regarding the supplementation of probiotics or not Fig. (**8b**). Group A is the most separated group. The separation between these three groups was correlated to 3 OTUs affiliated to the *Ruminococcaceae* family (undetermined species). Finally, a s-PLS DA was performed with Eof point for groups with probiotics or not Fig. (**8c**). The separation between groups is not so clear even though the cutoff used was lower (cutoff lowered to 0.75 instead of 0.95 for the 2 other ones). However, the separation between these groups was correlated to 2 OTUs affiliated to the *Barnesiella* genus (OTU_141 affiliated to *B. viscericola* 96.2% and OTU_195 affiliated to *Barnesiella spp.* 94.8%, OTU_183 affiliated to *Variovorax paradoxus* 100%) and to OTU_15 affiliated to *Lachnospiraceae* 100% (undetermined species). However, the relative abundance of these OTUs is less than 0.01%.

#### 3.6.4 sPLS Analysis: Relationship Between Bacterial Taxonomic Profiles and Liver Bio-Chemical Parameters and Growth Performances

As described for ileal samples, we studied the relationships between the bio-chemical plasma parameters and parameters and the OTU profile of the microbiota by sPLS. Fig. (**9**) is a graphical representation of the selected OTUs on the first two sPLS dimensions. In accordance with Table (**3**), bio-chemical and growth parameters were grouped together and positively correlated except for glycemia. Considering the OTUs, high number of them were positively correlated with bio-chemical and growth parameters. Most of them were members of *Clostridiales*, especially *Lachnospiracae* and *Ruminococcae*, already highlighted to be the most discriminant OTUs between experimental groups. Furthermore, *Lactobacillus* and *Pediococcu*s genera of *Lactobacillales* order were negatively correlated with these parameters.

## 4 DISCUSSION

In this study, high-throughput sequencing was used for the first time to identify the modulation of ileal and cecal microbiota, metabolism gene expression and bird performance after supplementation with *Lactobacillus salivarius* (isolated from ducks or as a mixture with other lactic acid bacteria) from hatching to the overfeeding period. *Lactobacillus* strains and other lactic acid bacteria have been described as increasing starch digestibility in chickens and energy harvested from food [28, 47]. Furthermore, *Lactobacillus salivarius* is the dominant species isolated from intestinal content in ducks and in geese in previous studies, as well as in our work [48, 49]. The use of *Lactobacillus* strains as probiotics has been very well described in broiler chickens, especially to stimulate an immune response, digestive health and growth performance [18, 24, 50]. In ducks, *Bacillus subtilis* is the strain most commonly used as a probiotic and few studies on the potential role of *Lactobacillus* as a probiotic have been reported [9, 51].

As previously described in ducks, geese and chickens, here, *Firmicutes*, *Proteobacteria* and *Bacteroidetes* are also dominant in all samples independently of sampling point, overfeeding, supplementation of probiotics or digestive contents [8-11, 27]. In the ileal samples, *Firmicutes* is the dominant phyla (more than 98% of sequences) and in particular the order *Clostridiales,* unlike in chickens where *Lactobacilliales* dominate [52]. *Proteobacteria* was the second most common phylum in our study, as well in Canada geese [53], graylag geese [10] and Muscovy (*Cairana moschata*) and mule ducks [8, 9]. This observation suggests that the bacterial digestive metabolism of chicken and waterfowl (both ducks and geese) could be quite different in terms of the ability to trigger a hepatic steatosis. In the cecal samples, even though the *Firmicutes* phylum was also dominant (up to 50%), *Bacteroidetes* (25%) and *Proteobacteria* (23%) were more abundant in our work in comparison with previous studies on ducks and geese [8-10]. Moreover, in chickens, geese and ducks, it has previously been evidenced that the intestinal microbiota is modulated by diet, environment and host genetics, which could partially explain the differences [8-11].

In our study, strong differences were observed between SP and Eof point in terms of microbial diversity in both the ileal and cecal samples, suggesting that age and overfeeding can modulate intestinal microbiota. Vasai *et al.* [9] showed little effect of overfeeding on the microbiota at the ceca level but in our study, we compared samples from ducks overfed with younger birds, which can probably explain the greater differences in microbial diversity in the ceca. The decrease in *Firmicutes* (order *Clostridiales*) associated with the increase in *Bacteroidetes* (order *Bacteroidiales*) and *Proteobacteria* has already been highlighted in a previous study in the lab [30]. Furthermore, in Pekin ducks, the most abundant bacteria (more than 95%) over the first 36 days were also *Firmicutes,* in particular, the *Clostridiales* order, as in our study [29]. Interestingly, in our work, at Eof point, a strong increase in the abundance of *Lactobacillus,* as described in geese and ducks, was observed but not linked to the supplementation of probiotics [8-10]. The abundance of *Lactobacillus* in overfeeding is high so we hypothesize that the supplementation did not yield statistical differences.

Overfeeding increased the relative abundance of *Lactobacillales* (essentially *Lactobacillus* genus) associated with a decrease in *Clostridiales,* as previously described in ducks and geese [8-10]. *Lactobacillus* strains are very well known as amylolytic bacteria and increase in pigs, rats and cattle with diets containing high levels of starch [54-56]. Furthermore, the *Lactobacillus* genus has been identified as increasing amylase activity in the small intestine in chickens [57]. Then, *Lachnospiracae* and *Ruminoccocae* were identified as most discriminant OTUs between experimental groups in both ileum and ceca. Moreover, these OTUs were respectively negatively and positively correlated with growth and bio-chemical parameters in ileum and ceca. Interestingly, these families were identified in previous works, as enriched in ceca of chickens with good FCR (food conversion ratio) and increased body weight [58, 59]. Then *Lactobacillus* were also correlated with body weight gain [58].

Overfeeding increased liver weight and fattening levels in the control group, in line with previous studies [8, 9, 14, 60]. However, while the body weight was affected by both probiotic supplementations during the first 28 days, other growth parameters (liver, fat and muscle weights) were not improved. Several works show an improvement in body weight and FCR (feed conversion ratio) and protection against pathogens [50, 61-63]. But other studies do not show any positive effect on the performance of chickens or ducks [9, 64, 65]. The differences obtained in these studies could be partially explained by the difference in strains and their concentrations, the methods of supplementation (feed, water, or invasive method) or the genetic strains of birds. Although liver weight was not affected by probiotic supplementation, slight differences in melting rate were observed after overfeeding. Next, the group with the *L. salivarius* supplementation only during the rearing period had a lower melting rate than the control group, suggesting that this strain could improve melting performance, which is a very important performance parameter for farmers and the industry. Nevertheless, it is now known that although the fatty liver melting rate is directly related to fatty liver weight, other parameters not yet identified are also implicated [66, 67].

Next, the plasma concentrations of glucose and triglycerides (TG) increased between SP and Eof point, which is quite consistent with the metabolic state of overfed ducks and geese, where significant changes occur [13, 14, 68, 69]. So as previously described in these studies, the overfeeding period strongly increased the TG and glucose plasma concentration in our study as well. However, probiotic supplementation had no effect on these parameters during both rearing and overfeeding periods. Moreover, at the end of the overfeeding period, the group with *L. salivarius* supplementation had a higher triglyceride concentration than group C at Eof point. Two known mechanisms during overfeeding may explain this difference. First, during overfeeding, *de novo* lipids synthesized in the liver are exported *via* the VLDL to the peripheral tissues *via* the blood circulation [13, 69]. The second mechanism is the lipid re-uptake of the liver at the end of overfeeding, as demonstrated by Tavernier *et al.* [14]. So the higher blood TG level can be explained either by a higher export or a lower lipid re-uptake, or both. Interestingly, overfeeding had a huge impact on host metabolic gene expression, as previously described [14, 60]. Next, genes implicated in *de novo* lipogenesis (*Fasn, DGAT2*), in Fatty acid transport (*FABP4*), increased strongly. A modulation of the energy balance enhanced by intestinal microbiota, as demonstrated by Bäckhed *et al.* [1], could be also related to the change in plasma TG and glucose concentrations. Several studies show that in mammals, the microbiota triggers the storage of triglycerides through the suppression of the Fiaf factor, a lipoprotein lipase (LPL) circulating inhibitor [1, 70, 71]. In ducks, the LPL activity correlates positively with a higher storage in peripheral tissues instead of fat storage in the liver [13, 72, 73]. Interestingly, the expression of the Fiaf factor increases significantly during the overfeeding period according to the decrease in LPL activity during overfeeding in mule and Muscovy ducks [13, 72, 73]. Changes observed in intestinal microbiota after overfeeding allow us to partially explain metabolic changes in ducks *via* the FIAF factor expression. Furthermore, in mice, the implication of the intestinal microbiota has been identified in susceptibility to hepatic steatosis, as described in mice regarding NASH susceptibility [74]. Moreover, hepatic steatosis in mice is generally associated with an increase in *Firmicutes* [74, 75] while a significant increase in the *Clostridiales* order is associated with metabolic protection [75]. Here, *Firmicutes* remained stable, but *Lactobacilliales* increased whereas *Clostridiales* decreased on the order level.

Moreover, immune response was also modulated during the overfeeding period, as previously described in geese [10]. These authors showed that the complement system, part of the inflammatory system, was suppressed during overfeeding and partially explained it by an increase in blood lactic acid from enriched *Lactobacillus*. Here, *LITAF* (responding to LPS, a component of the gram-negative wall) as well as IL-8 gene expression decreased when *PPARγ* increased after overfeeding, in line with the decrease in *Campylobacter* observed. Furthermore, supplementation with *L. sakei* in ducks leads to a decrease in *Enterobacteria* [9]. Other probiotic strains such as *Enterococcus faecalis* are also able to down-regulate *PPARγ* activity and IL-10 levels in humans [76]. Furthermore, in chickens, *Lactobacillus* in diet protects birds against coccidiosis by enhancing immune stimulation [23].

In our study, probiotic supplementation did not affect metabolic gene expression during the rearing or overfeeding periods. Probiotic supplementation allowed us to show a slight effect on immune response modulation during the overfeeding period, more specifically for the *Litaf* gene. The *Litaf* gene encodes for a transcription factor activated in response to the presence of lipopolysaccharide. As mentioned above, the *Lactobacillus* and *Enterococcus* genera are known to modulate the immune response. The presence of these two genera in probiotic B could therefore explain the lower pro-inflammatory response in this group showed by a decrease in *Litaf* expression. Probiotic supplementation had no statistical effect on microbial population, either in the ileum or the caeca contents at phylum or order levels during the rearing period. In the same way, probiotic supplementation had a slight effect on bacterial communities during the overfeeding period.

## CONCLUSION

To conclude, our results confirm the significant changes to metabolism gene expression and microbial diversity triggered by overfeeding, but not so much by probiotic supplementation. Interestingly, anti-inflammatory response seems to be decreased in overfed ducks and probably explained by changes in microbial composition. This work probably partially explains the tolerance to hepatic steatosis in ducks. The identification of protective factors could also offer therapeutic clues against hepatic steatosis in humans.

## Figures and Tables

**Fig. (3):**
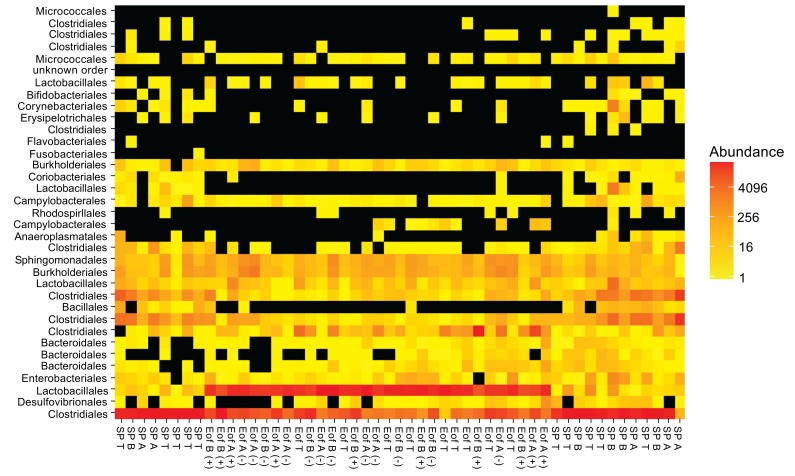
Heatmap representation of ileal microbiota at family level. Samples and OTUs were clustered using nMDS ordination with the Bray-Curtis ecological distance method. The different families are indicated at the level of the order. Samples were indicated according to experimental group.

**Fig. (4):**
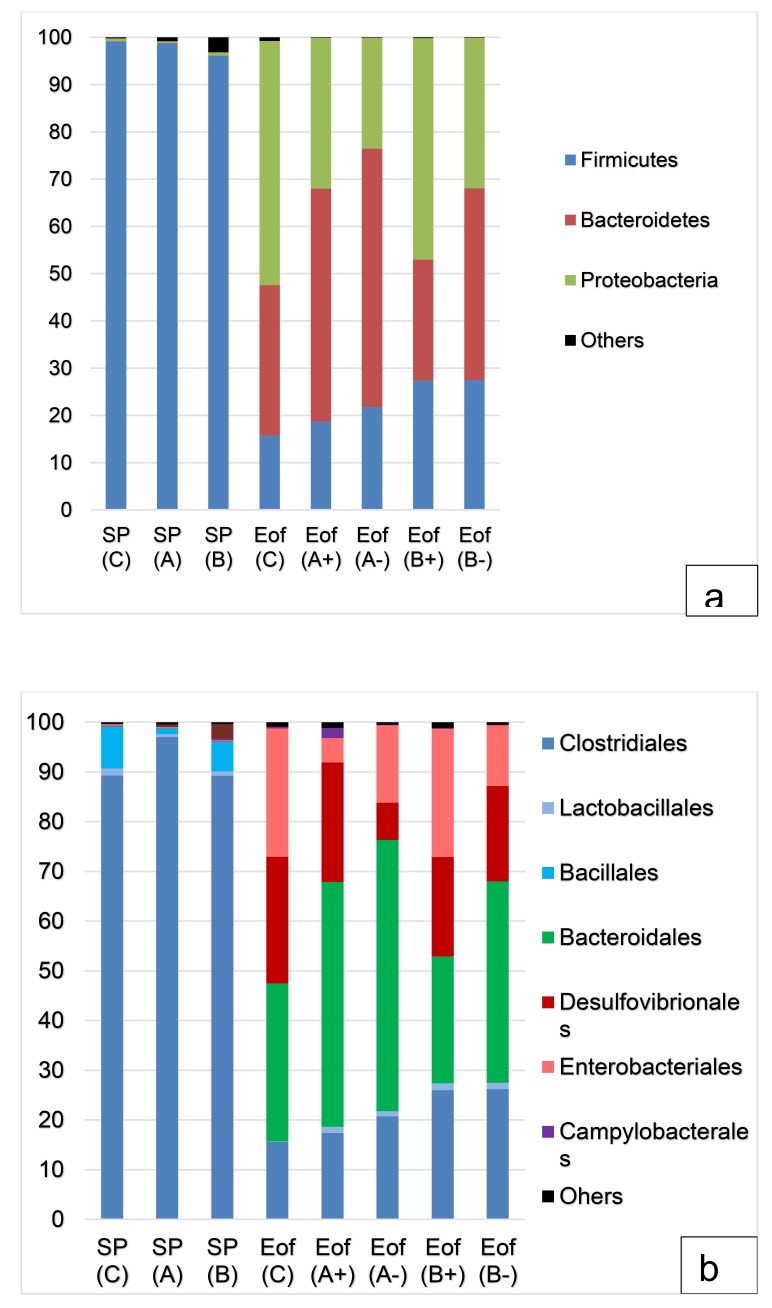
Relative abundance (%) evaluated at the phylum (a) and order (b) levels of main bacterial groups in the ileum before (Bof) and after (Eof) overfeeding according to experimental group.

**Fig. (5):**
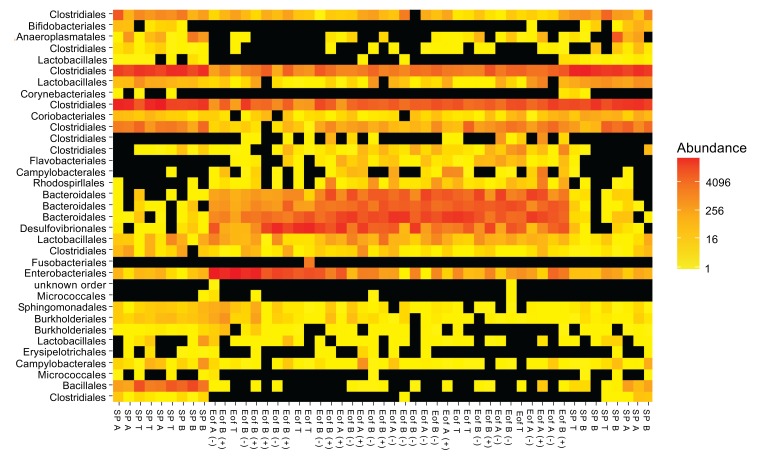
Heatmap representation of cecal microbiota at family level. Samples and OTUs were clustered using nMDS ordination with the Bray-Curtis ecological distance method. The different families are indicated at the level of the order. Samples were indicated according to experimental group.

**Fig. (6):**
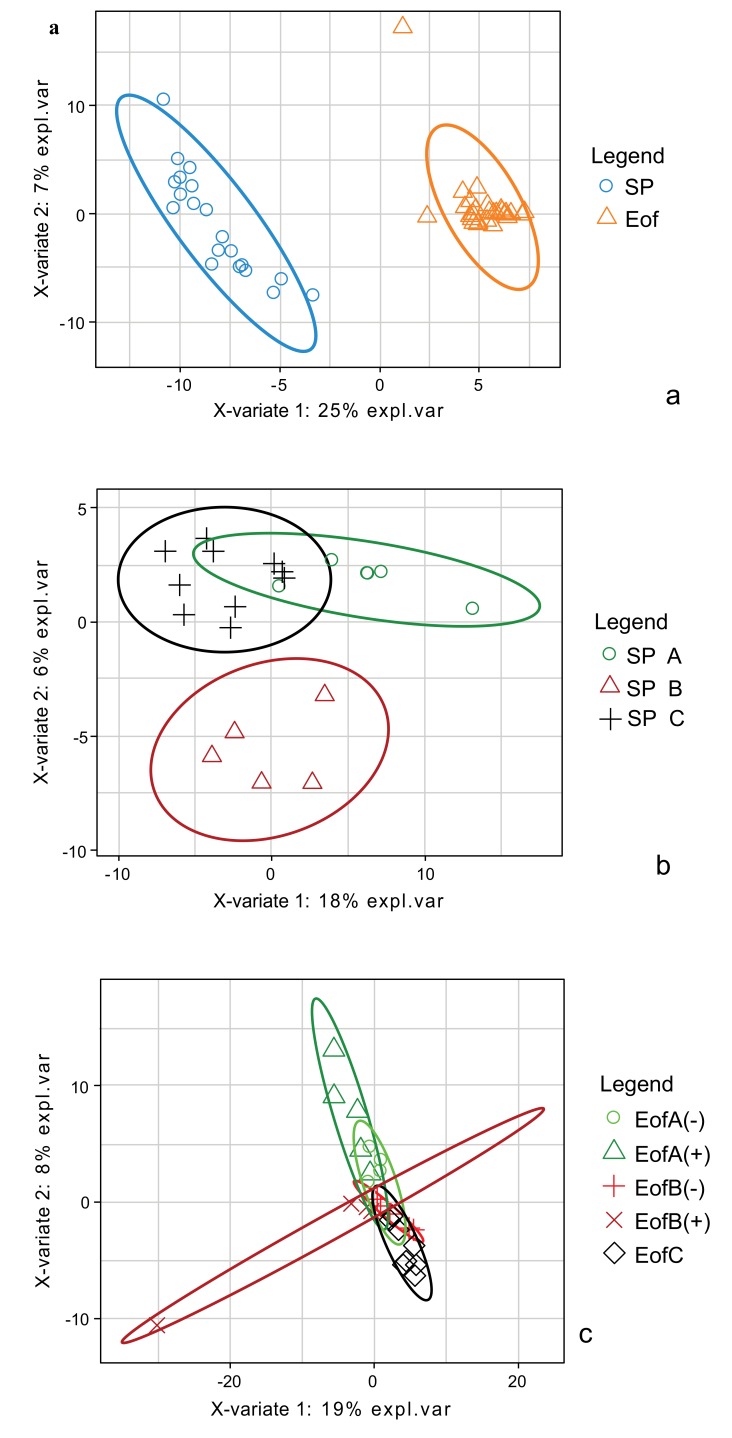
Sparse Partial Least Square Discriminant Analysis (sPLS-DA) of the ileal microbial community (a) before and after overfeeding independently of experimental 2 group, (b) between the three experimental groups during the rearing period, (c) between the five experimental groups after the overfeeding period according to the first two explanatory variables. Control groups before and after overfeeding are represented in black while the experimental groups with probiotic A are represented in green and groups with probiotic B in red.

**Fig. (7):**
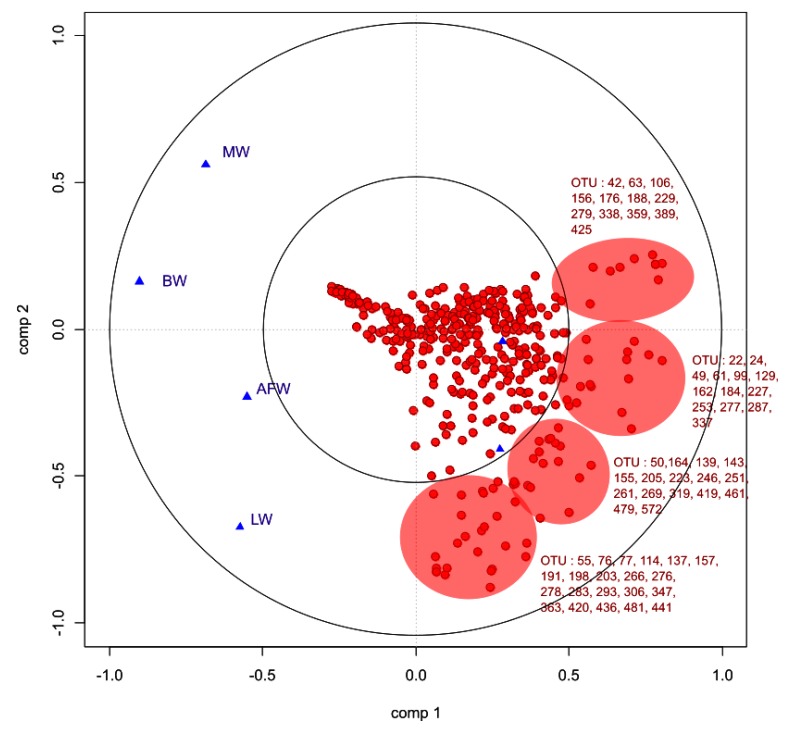
sPLS variables representation of the liver biochemical parameters and growth parameters (blue) and the selected bacterial OUT in ileal microbiota (red) on the first two sPLS dimensions. *Variable lying outside the small correlation circle are highly correlated. Variables that cluster together are correlated.*

**Fig. (8):**
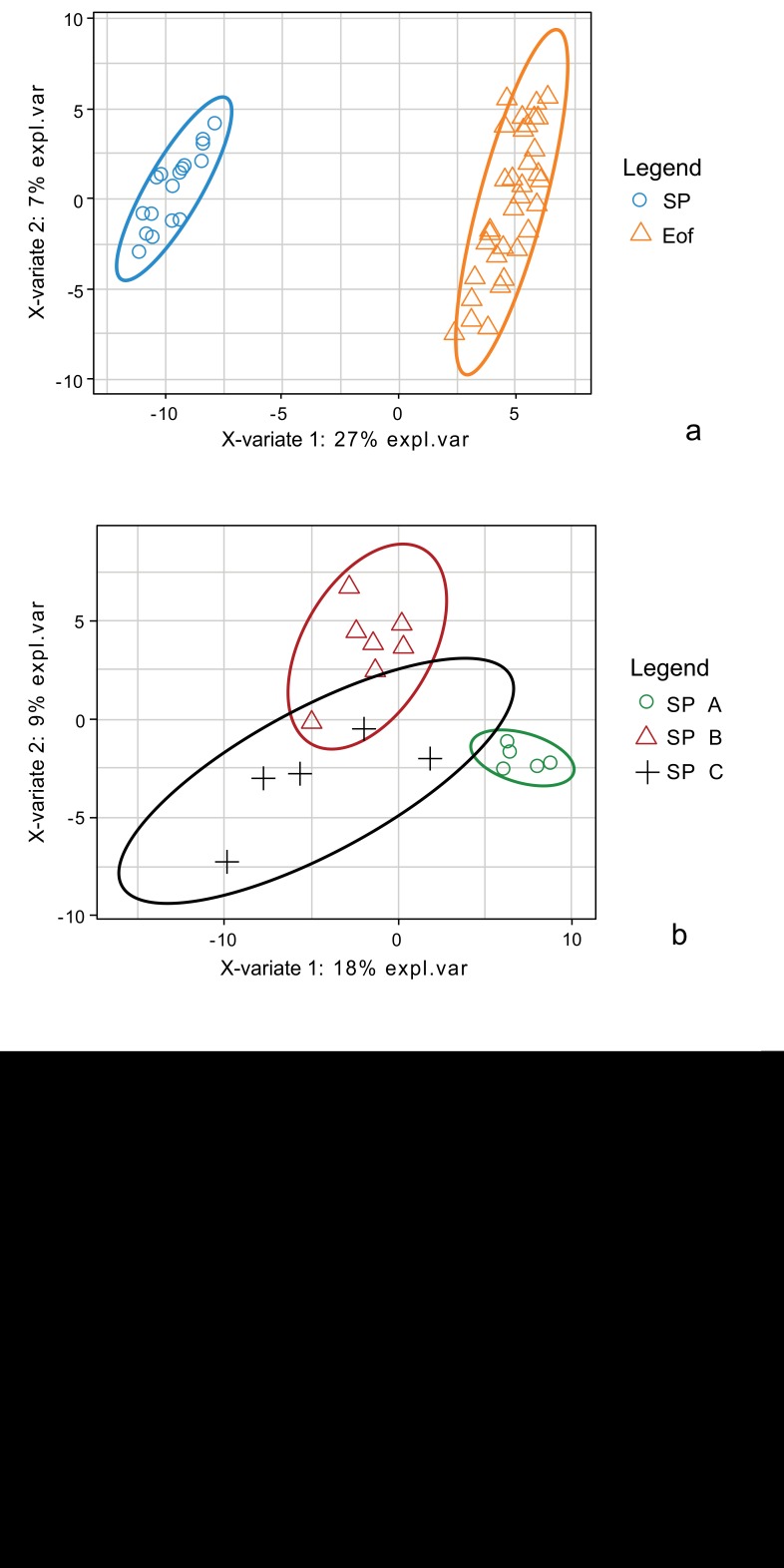
Sparse Partial Least Square Discriminant Analysis (sPLS-DA) of the cecal microbial community (a) before and after overfeeding independently of experimental group, (b) between the three experimental groups during the rearing period, (c) between the five experimental groups after the overfeeding period according to the first two explanatory variables. Control groups before and after overfeeding are represented in black while experimental groups with probiotic A are represented in green and groups with probiotic B in red.

**Fig. (9):**
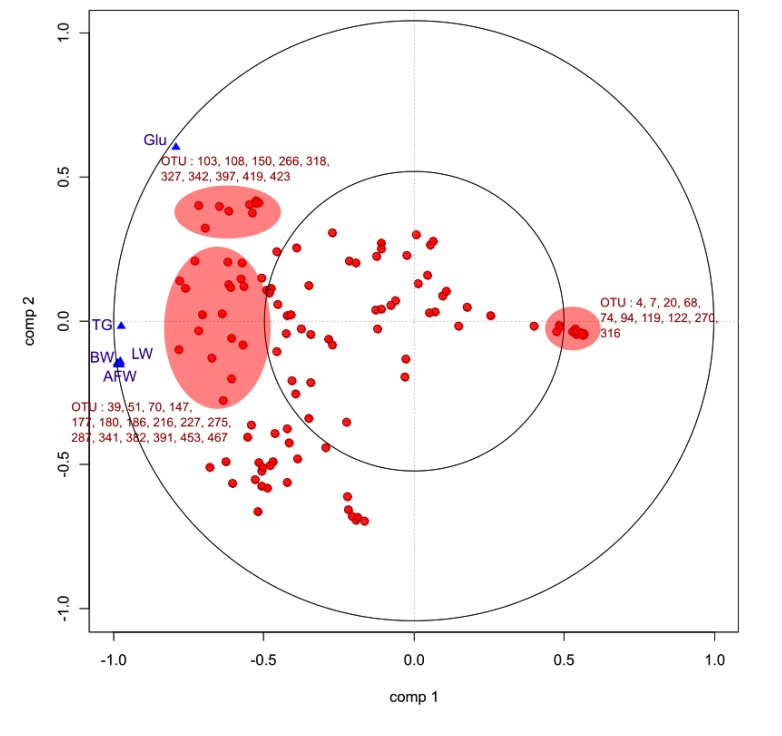
sPLS variables representation of the liver biochemical parameters and growth parameters (blue) and the selected bacterial OUT in cecal microbiota (red) on the first two sPLS dimensions. Variable lying outside the small correlation circle are highly correlated. Variables that cluster together are correlated.

**Table 1:** Ingredients and main nutrient composition of the experimental diets, expressed in percentage of raw feed unless overfeeding diet expressed in percentage of DM.

Ingredients		Starter diet	Grower diet	Overfeeding diet (% of Dry Matter)
	Wheat	39,9	35	
	Corn	20,1	15	98
	Others cereals or derivatives	13,6	19,5	
	Oilcakes	22,9	22,65	
	Premix and vitamin	3	2,6	2
Nutrients				
	ME MJ/kg	11,43	11,37	13,9
	Starch	40,2	47,3	75,9
	Humidity	12.23	12.07	
	CP	17.50	15.51	8,91
	Cellulose	5.14	6.01	2,77
	Ashes	5.50	4.97	4,99

**Table 2:** Primers used for determination of mRNA levels.

Gene (Name and Symbol)	References	Primer Sequence 5′-3′	Product Size (bp)
Apolipoprotein B	[16]	TCTCACCGTGACTTGAGTGC	137
ApoB		TCCCAGCAGAAGGTGAAGAT	
Fatty acid binding protein 4	[16]	AATGGCTCACTGAAGCAGGT	143
FABP4		TGGCTTCTTCATGCCTTTTC	
Fatty acid synthase	[16]	TGAAGAAGGTCTGGGTGGAG	97
FAS		CTCCAATAAGGTGCGGTGAT	
Fatty acid translocase/cluster of	[16]	AGTTTGCCAAAAGGCTTCAA	228
Differentiation 36 FAT/CD36		CGAGGAACACCACAGAACCT	
Peroxisome proliferator activated	[16]	CCCAAGTTTGAGTTCGCTGT	196
Receptor Gamma PPARG		GCTGTGACGACTCTGGATGA	
Glucose transporter 2	[67]	GGAGTTGACCAACCCGTTTA	242
GLUT2 (SLC2A2)		CCCACCTCGAAGAAGATGAC	
lipopolysaccharide-induced TNF factor	By this study	GTCTGCACCACCTTCTTATGAG	163
LITAF		CCGTCTGAACTGTAACGGG	
angiopoitein-likeprotein 4	By this study	CCCTTCAAAGTCTACTGCGA	136
FIAF/ANGPTK-4		CAGAAGTCACCATGAAGGTCTC	
Interleukine 8	By this study	GAAATCATAGCTACTCTGAAGGAC	112
IL8		CAGAATTGCCTTTACGATCCG	
Reference genes			
Actin B	[16]	CCAGCCATCTTTCTTGGGTA	141
ActB		ATGCCTGGGTACATTGTGGT	
glyceraldehyde 3-phosphate dehydrogenase	[16]	CAGAGGACCAGGTTGTCTCC	146
(GAPDH)		CACCACACGGTTGCTGTATC	

**Table 3:** Zootechnical results (in g) and plasma of glucose (Glu) and tryglicerides (TG) concentrations according to experimental groups^1^.

Zootechnical parameters	Group C Bof	Group A Bof	Group B Bof	Group C Eof	Group A+ Eof	Group A- Eof	Group B+ Eof	Group B- Eof	*P*value
Body weight (g)	4 306+/-75a	4 310+/-82a	4299+*-64a	6261±97b	6382±108b	6330±112b	6441±99b	6268±157b	***
Liver weight (g)	93+/-5a	90+/-4a	93+/-3a	628±21b	593±27b	564±27b	605±32b	584±34b	***
Fat weight (g)	57+/-8a	51+/-5a	59+/-7a	151±5b	153±6b	152±8b	162±6b	157±9b	***
Muscle weight (g)	313+/-8	317+/-9	310+/-13	315±5	318±8	317±6	314±8	316±11	N.S
Melting rate (%)	N.D	N.D	N.D	19,6±1,2a	13,7±2,3ab	12,7±1,7b	17,9±2,5ab	14,8±1,9ab	*
Feed consumption (g)	21 969	21 769	21 636	8722±18	8714±34	8662±62	8721±27	8541±154	N.S
Glu (g/L)	1.99+/-0.71a	1.97+/-0.21a	1.96+/-0.30a	2,94±0,84b	2,80±0,81b	3,14±0,65b	2,95±0,51b	3,39±1,14b	***
TG (mmol/L)	1.43+/-0.25c	1.19+/-0.43c	1.34+/-0.23c	4,76±1,37a	5,86±1,32b	5,24±1,39a,b	5,51±1,10a,b	4,51±0,68a	***

^1^The values are expressed as means ± standard error of the mean (SEM). a, b, c, Means in the same row with unlike superscripts differ (P < 0.05).

**Table 4:** Relative expression of genes implicated in metabolic and immune response in liver, muscle and jejunum mucosa according to experimental groups. ^2^

Organ	Gene	Group A SP point	Group B SP point	Group C Eof point	Group A+ Eof point	Group A- Eof point	Group B+ Eof point	Group B- Eof point	*P*value
Liver									
Lipid metabolism	FAS	1±0a	1±0a	717±312b	334±95b	439±148b	410±189b	604±273b	*
	FABP4	3±1a	1±0a	177±77b	58±10b	87±21b	105±27b	57±26b	**
	FAT/CD36	2.84±2.23	0.94±0.43	0.04±0.01	0.04±0.01	0.07±0.02	0.05±0.01	0.07±0.03	N.S
	ApoB	n.d	n.d	n.d	2,4±1,7	2,3±1,7	8,6±4,6	7,1±6,4	N.S
Glucid metabolism									
	GluT2	n.d	n.d	n.d	1.6±0.1	1.5±0.1	1.3±0.1	1.4±0.1	N.S
Muscle									
Lipid metabolism	FAS	n.d	n.d	n.d	1.07±0.56	1.19±0.49	0.60±0.16	0.69±0.23	N.S
Jejunal mucosa									
Immune response	PPARG	0,9±0,3a	2,2±1,7a	4,8±0,4b	4,1±0,6b	3,8±0,7b	3,2±0,8b	4,4±0,7b	**
	LITAF	0.97±0.30a	0.68±0.21a	0.42±0.09b	0.55±0.11b	0.47±0.05b	0.27±0.07c	0.39±0.07bc	**
	IL8	0,8±0,1	1,1±0,2	n.d	n.d	n.d	n.d	n.d	N.S
Metabolism regulation									
	FIAF	1.03±0.08a	1.16±0.25a	3.69±0.53b	4.50±0.51bc	3.51±0.75bc	2.72±0.89ab	5.23±0.59d	**

^2^The values are expressed as means ± standard error of the mean (SEM) of 2-∆∆Ct with group C at SP point as reference group. a, b, c, d Means in the same row with unlike superscripts differ (P < 0.05).

**Table 5:** Estimators of diversity in ileum and ceca of ducks according to experimental groups. ^3^

Alpha diversity parameters	Group C SP point	Group A SP point	Group B SP point	Group C Eof point	Group A+ Eof point	Group A- Eof point	Group B+ Eof point	Group Eof point B-	*P*value
Ileon									
Observed	133±16a,b	167±26a	158±19a	75±6b	90±17a	77±10b	81±21b	70±2b	***
Chao1	162±18a,b	199±28a	192±22a	96±9b	114±30a,b	99±15b	93±21b	89±3b	***
Shannon	0,5±0,1a	1,3±07ab	0,7±0,3ab	1,8±0,1b	2,0±0,2b	1,8±0,1b	1,5±0,3b	1,8±0,1b	***
InvSimpson	1.2±0.1	5.5±4.3	1.4±0.2	3.9±0.3	5.1±0.9	4.1±0.6	3.3±0.8	3.6±0.4	N.S
Ceca									
Observed	199±24	227±2	214±8	161±28	218±43	224±18	203±24	228±17	N.S
Chao1	223±24	246±6	232±8	209±30	255±41	266±21	234±21	269±17	N.S
Shannon	3,6±0,2a,b	4,1±0,1a	3,8±0,2a,c	2,1±0,5b	2,7±0,4a,b	2,7±0,3a,b	2,6±0,3b,c	2,9±0,3a,b	***
InvSimpson	17.2±2.7a,b	30.0±5.0a	27.0±6.7a	6.9±3.1b	7.8±2.3b	7.8±2.3b	7.4±2.0b	9.1±2.0b	***

^3^The values are expressed as means ± standard error of the mean (SEM). a, b, c, Means in the same row with unlike superscripts differ (P < 0.05).
